# Perturbation of the Hematopoietic System during Embryonic Liver Development Due to Disruption of Polyubiquitin Gene *Ubc* in Mice

**DOI:** 10.1371/journal.pone.0032956

**Published:** 2012-02-29

**Authors:** Kwon-Yul Ryu, Hyejin Park, Derrick J. Rossi, Irving L. Weissman, Ron R. Kopito

**Affiliations:** 1 Department of Life Science, University of Seoul, Seoul, Republic of Korea; 2 Immune Disease Institute, Harvard Stem Cell Institute, and the Department of Pathology, Harvard Medical School, Boston, Massachusetts, United States of America; 3 Department of Pathology, Stanford University School of Medicine, Stanford, California, United States of America; 4 Department of Biology, Stanford University, Stanford, California, United States of America; Emory University, United States of America

## Abstract

Disruption of the polyubiquitin gene *Ubc* leads to a defect in fetal liver development, which can be partially rescued by increasing the amount of ubiquitin. However, it is still not known why *Ubc* is required for fetal liver development and the nature of the defective cell types responsible for embryonic lethality have not been characterized. In this study, we assessed the cause of embryonic lethality with respect to the fetal liver hematopoietic system. We found that *Ubc* was highly expressed in the embryonic liver, and the proliferation capacity of fetal liver cells was reduced in *Ubc^−/−^* embryos. Specifically, *Ubc* was most highly expressed in hematopoietic cells, and the proliferation capacity of hematopoietic cells was significantly impaired in *Ubc^−/−^* embryos. While hematopoietic cell and hematopoietic stem cell (HSC) frequency was maintained in *Ubc^−/−^* embryos, the absolute number of these cells was diminished because of reduced total liver cell number in *Ubc^−/−^* embryos. Transplantations of fetal liver cells into lethally irradiated recipient mice by non-competitive and competitive reconstitution methods indicated that disruption of *Ubc* does not significantly impair the intrinsic function of fetal liver HSCs. These findings suggest that disruption of *Ubc* reduces the absolute number of HSCs in embryonic livers, but has no significant effect on the autonomous function of HSCs. Thus, the lethality of *Ubc^−/−^* embryos is not the result of intrinsic HSC failure.

## Introduction

Ubiquitin (Ub) is a small, highly conserved eukaryotic protein that plays a crucial role in diverse cellular signaling pathways, including targeting proteins for proteasomal degradation [1], [2], [3]. Inside cells, Ub exists in a dynamic equilibrium between free Ub and monomeric/polymeric Ub-substrate conjugate pools [4], [5]. The Ub conjugation reaction is mediated by a series of enzymes E1-E3, and the deconjugation reaction is mediated by isopeptidases or deubiquitylating enzymes, during which most Ub is recycled back to the free Ub pool [6], [7]. It is believed that maintaining cellular steady-state Ub levels is important for their function and survival [8], [9], [10]. Although Ub is a unique protein, it is encoded by two different classes of ubiquitin genes; constitutively expressed monomeric Ub ribosomal fusion genes and stress-regulated polyubiquitin genes [11], [12]. Under stress or even normal conditions, the contribution of polyubiquitin genes towards total cellular Ub levels is very significant [13], [14].

In mammals, there are two polyubiquitin genes, *Ubb* and *Ubc*, and disruptions of these genes in mice have been shown to exhibit various phenotypes [13], [14], [15], [16], [17]. Disruption of *Ubb* reduced Ub levels in the gonads and hypothalamus, which resulted in infertility, hypothalamic neurodegeneration, metabolic abnormalities, and impaired energy and sleep homeostasis [14], [15], [16], [17]. On the other hand, disruption of *Ubc* resulted in embryonic lethality with defective fetal liver development [13]. Although ectopic expression of Ub partially rescued the *Ubc^−/−^* phenotypes by delaying the onset of lethality, the precise mechanism underlying the cause of *Ubc^−/−^* embryonic lethality is still unknown.

In a study that used mouse embryonic fibroblasts (MEFs) isolated from *Ubc^−/−^* embryos, the phenotypes of *Ubc^−/−^* MEFs including reduced proliferation and delayed cell-cycle progression were found to be completely rescued by increasing cellular Ub levels [13]. However, under stress conditions, ectopic expression of Ub was not sufficient to increase the cellular Ub levels observed in wild type cells under stress, resulting in the failure of rescuing the phenotypes of *Ubc^−/−^* MEFs. Therefore, the phenotypes of *Ubc^−/−^* MEFs are a direct consequence of reduced cellular Ub levels. In addition, we previously showed that the contribution of *Ubc* toward total Ub levels is highest in liver among all other tissues investigated in adult mice [13]; therefore, it is highly likely that the defective fetal liver development is closely related to the reduced cellular Ub levels in *Ubc^−/−^* embryonic liver.

Here, as was observed in *Ubc^−/−^* MEFs, we found that fetal liver cells exhibit reduced proliferation, presumably due to the reduced cellular Ub levels in fetal liver. Rapid enlargement of the fetal liver during the midgestation period is important because the fetal liver becomes the primary site of hematopoiesis at embryonic days (E) 11–11.5, during which hematopoietic stem cells (HSCs) seed the liver from the aorta-gonad mesenepheros (AGM) region [18]. Through hematopoiesis, all the blood cell types are generated including myeloid and lymphoid lineages [19], therefore it is an essential process for survival. It has also been known that the cycling status of fetal liver HSCs is higher than that in adult bone marrow (BM) stem cells, which are largely quiescent [20]. However, here we demonstrate by non-competitive and competitive reconstitution methods that cell autonomous hematopoietic function in the *Ubc^−/−^* embryonic liver is essentially intact. These findings show that *Ubc^−/−^* embryonic lethality is not directly caused by the impaired function of HSCs themselves, but rather by autonomous insufficiency in *Ubc^−/−^* liver function to support hematopoiesis.

## Results

### Reduced proliferation and increased apoptosis in *Ubc^−/−^* fetal liver cells

**Figure 1:**
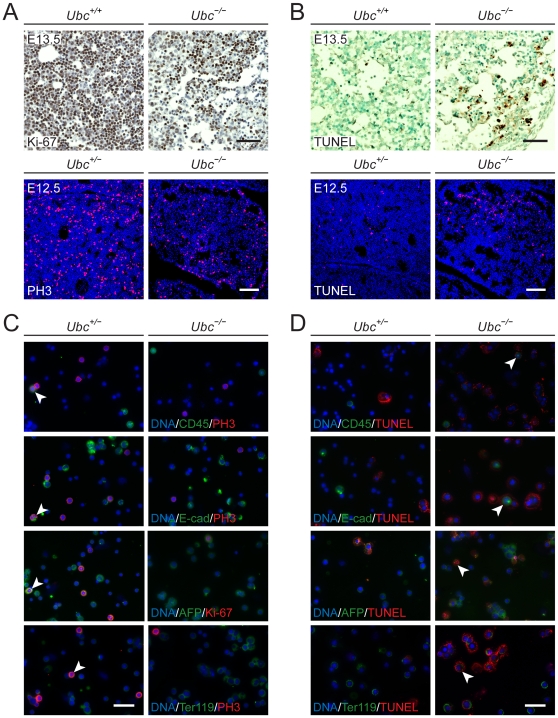
Reduced proliferation and increased apoptosis in *Ubc^−/−^* fetal liver cells. (A) (Upper panel) Paraffin-embedded liver sections were prepared from wild-type (*Ubc^+/+^*) and *Ubc^−/−^* embryos at E13.5 and stained with the proliferation marker, Ki-67. (Lower panel) Frozen liver sections were prepared from *Ubc^+/−^* and *Ubc^−/−^* embryos at E12.5 and stained with the proliferation/mitotic marker, PH3 (red), and DNA was visualized using TO-PRO-3 iodide (blue). *Ubc^+/−^* embryos also served as controls because they had no phenotypic difference relative to wild-type embryos. (B) (Upper panel) TUNEL assay of paraffin-embedded E13.5 embryonic liver sections. TUNEL-positive cells were rare in *Ubc^+/+^* embryonic liver sections, but were observed focally in *Ubc^−/−^* embryonic liver sections. (Lower panel) TUNEL assay of frozen E12.5 embryonic liver sections (red), with DNA visualization using TO-PRO-3 iodide (blue). (C). Cytospin slides were prepared from *Ubc^+/−^* and *Ubc^−/−^* embryos at E13.5 and stained with CD45 (pan-hematopoietic marker), E-cadherin (epithelial cell marker), AFP (hepatocyte marker), and Ter119 (erythroid cell marker) in combination with a proliferation marker (PH3 or Ki-67) and DNA was visualized with DAPI. Examples of proliferating cells that were also positive for cell-type specific markers were indicated by arrowheads. (D) Fetal liver cells on cytospin slides were subjected to the double-labeling fluorescence TUNEL assay with appropriate markers. Examples of apoptotic cells that were also positive for cell-type specific markers were indicated by arrowheads. All data are representative images from three different embryos per genotype. Scale bars, 50 µm (upper panels in (A/B) and all panels in (C/D)); 200 µm (lower panels in (A/B)).

We have previously demonstrated that targeted disruption of polyubiquitin gene *Ubc* leads to embryonic lethality between embryonic days (E) 12.5 and 14.5, which is most likely due to the defect in fetal liver development [13]. Although we were able to partially rescue the lethal phenotype of *Ubc^−/−^* embryos by ectopic expression of Ub, the exact cause of embryonic lethality is still unknown. At stages E11–11.5, the size of the liver rapidly increases because it becomes the principal source of hematopoietic activity. This suggests that fetal liver cells need to undergo rapid proliferation during the midgestation period to reach a dense and homogenous architecture at E13.5. In fact, we have shown that the size of the fetal liver is smaller in *Ubc^−/−^* embryos when compared to wild type (*Ubc^+/+^*) at E13.5 [13]. To determine whether the reduced size of *Ubc^−/−^* fetal liver was due to the reduced proliferation of fetal liver cells, sagittal liver sections from E12.5 and E13.5 embryos were generated and stained with a proliferation marker, Ki-67 or phospho-histone 3 (PH3) (Figure 1A). As expected, we observed a marked reduction in the proliferation of *Ubc^−/−^* fetal liver cells, which was demonstrated using two different proliferation markers (Ki-67 and PH3). In addition, not only was reduced cellularity apparent, but also an altered liver architecture with dissociated parenchymal cells were observed in *Ubc^−/−^* fetal liver (Figure 1A, upper panel). Therefore, to determine whether apoptosis increased in *Ubc^−/−^* fetal liver, liver sections from E12.5 and E13.5 embryos were analyzed using the TUNEL assay (Figure 1B). Although TUNEL-positive cells were rare in wild-type or *Ubc^+/−^* liver sections, positive cells were observed focally in *Ubc^−/−^* liver sections, especially where the liver structure was altered.

To identify the cell types that were affected in proliferation and apoptosis due to deletion of *Ubc*, we then processed fetal liver cells from E13.5 embryos to prepare cytospin slides attached with equal number of fetal liver cells. We stained fetal liver cells with CD45 (pan-hematopoietic marker), E-cadherin (epithelial cell marker), AFP (hepatocyte marker), and Ter119 (erythroid cell marker) in combination with a proliferation marker (Figure 1C) or with an apoptotic cell detection using the TUNEL assay (Figure 1D). We found that the number of CD45, E-cadherin, AFP, and Ter119-positive cells were not largely distinguishable between *Ubc^+/−^* and *Ubc^−/−^* fetal liver cells. Based on our data, all cell types that we investigated exhibited reduced proliferation in *Ubc^−/−^* embryonic livers (Figure 1C). Intriguingly, we found that Ter119-positive *Ubc^−/−^* fetal liver cells were more apoptotic than any other cell types that we investigated (Figure 1D). Taken together, it seems that, due to deletion of *Ubc*, all cell types were affected in proliferation and Ter119-positive cells were affected most in apoptosis. Therefore, *Ubc* may be necessary for fetal liver cell proliferation and may protect cells from undergoing apoptosis. These results may suggest that *Ubc* expression is required in an attempt to increase cellular Ub levels in these apoptotic cells. It is also possible that *Ubc* may be upregulated as a stress response in apoptotic cells, although we cannot exclude the possibility that Ub deficiency in a *Ubc^−/−^* background may lead to cellular apoptosis.

### Ub protein and *Ubc* expression levels are high in fetal liver

**Figure 2:**
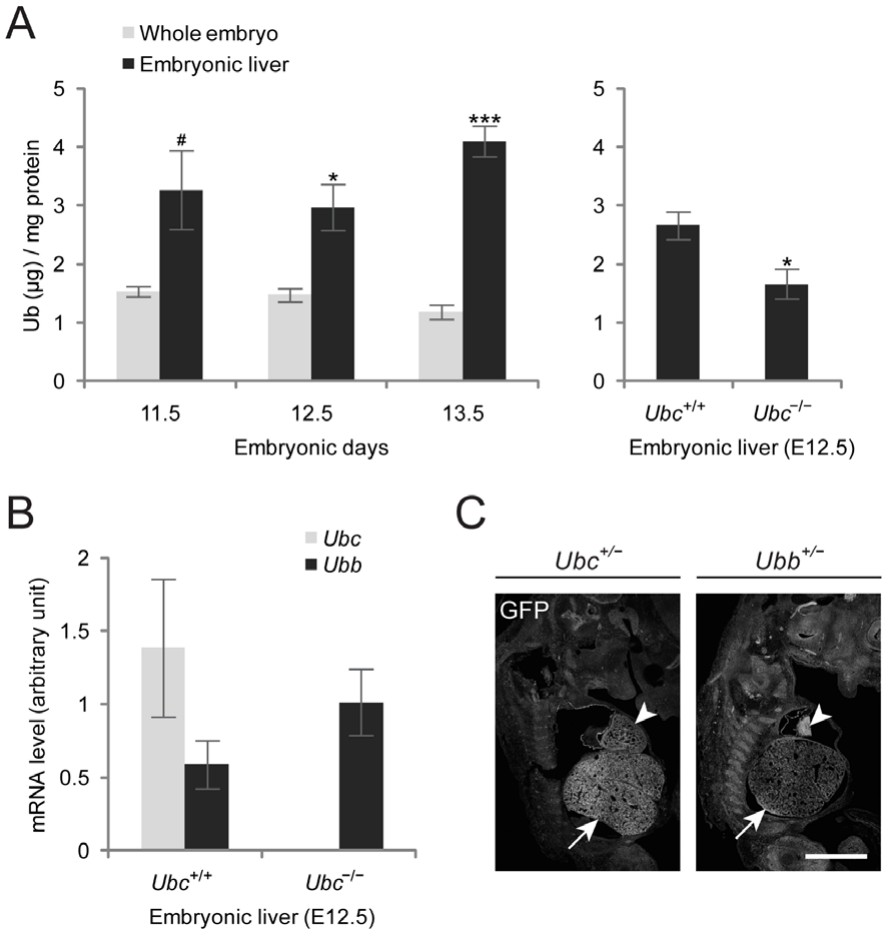
Increased Ub protein and *Ubc* expression levels in fetal liver. (A) (Left panel) Total Ub levels in whole embryos and embryonic livers during midgestation period from E11.5 to E13.5. Tissue lysates from wild-type embryos (*n* = 3) or embryonic livers (*n* = 3) at different embryonic days were treated with Usp2-cc and subjected to indirect competitive ELISA. (Right panel) Total Ub levels in *Ubc^+/+^* and *Ubc^−/−^* embryonic livers (*n* = 3 each) at E12.5. Ub levels in *Ubc^−/−^* embryonic livers were significantly reduced (by about 40%). (B) *Ubc* and *Ubb* mRNA levels in *Ubc^+/+^* and *Ubc^−/−^* embryonic livers (*n* = 3 each) at E12.5. Total RNA was isolated from embryonic livers and *Ubc* and *Ubb* mRNA levels were measured by quantitative real-time RT-PCR and normalized to 18S rRNA levels. No *Ubc* mRNA was detected in *Ubc^−/−^* embryonic livers and slight compensation by *Ubb* mRNA in *Ubc^−/−^* embryonic livers was observed. (C) High *Ubc*, but not *Ubb*, expression in embryonic livers. *Ubc* and *Ubb* expression in embryos at E12.5 was monitored by direct visualization of GFP fluorescence in *Ubc^+/−^* and *Ubb^+/−^* embryos, respectively. Arrows indicate embryonic livers and arrowheads indicate embryonic hearts, in which both *Ubc* and *Ubb* are highly expressed. All data are expressed as the means ± SEM from the indicated number of samples. ^#^ *P*<0.1; ^*^ *P*<0.05; ^***^ *P*<0.001 *vs.* Ub levels in whole embryos (A) or wild-type (*Ubc^+/+^*) embryonic livers (B). Scale bar, 1 mm.

To investigate how cellular Ub levels in the fetal liver are affected during embryonic development, we determined total Ub levels in whole embryos and embryonic livers by indirect competitive ELISA, in which all forms of Ub was converted to monomeric Ub using a Ub-specific protease (Usp2-cc) and the monomeric Ub was quantified (Figure 2A, left panel) [21]. We found that total Ub levels in both whole embryos and embryonic livers were well maintained throughout the midgestation period from E11.5 to E13.5, with significantly higher Ub levels in embryonic livers. However, a loss of *Ubc* reduced total Ub levels by approximately 40% in E12.5 embryonic liver (Figure 2A, right panel). In accordance with cellular Ub levels during embryonic development, *Ubc* mRNA levels, which were determined by quantitative real-time RT-PCR, did not change significantly during the midgestation period (data not shown). Although it was expected that the other polyubiquitin gene *Ubb* would be upregulated to compensate for the loss of *Ubc* in *Ubc^−/−^* fetal liver, because of the lower levels of *Ubb* than *Ubc* in fetal liver as well as the fact that the Ub-coding potential of *Ubb* was about half that of *Ubc* (9 Ubs per *Ubc* transcript *vs.* 4 Ubs per *Ubb* transcript), it seems that its contribution to increase the total Ub levels was quite minimal (Figure 2B). In accordance with high Ub levels in embryonic livers, the spatial distribution of *Ubc* expression in *Ubc^+/−^* embryos, which was determined by direct visualization of GFP fluorescence from a GFP-puro^r^ fusion protein knocked in to the *Ubc* locus, clearly indicated that *Ubc* was highly expressed in the embryonic liver (Figure 2C, indicated by an arrow). In contrast, *Ubb* was not expressed at high levels in the *Ubb^+/−^* embryonic liver, and was comparable to other tissues (Figure 2C, indicated by an arrow), although both *Ubb* and *Ubc* were highly expressed in the embryonic heart (Figure 2C, indicated by arrowheads). Therefore, given the fact that the contribution of *Ubc* to the total Ub levels in liver was relatively higher than in other tissues [13], high *Ubc* expression in the embryonic liver seems to be responsible for the high Ub levels in the embryonic liver.

### *Ubc* expression levels are high in hematopoietic cells and their proliferation capacity is impaired in *Ubc^−/−^* fetal liver

**Figure 3:**
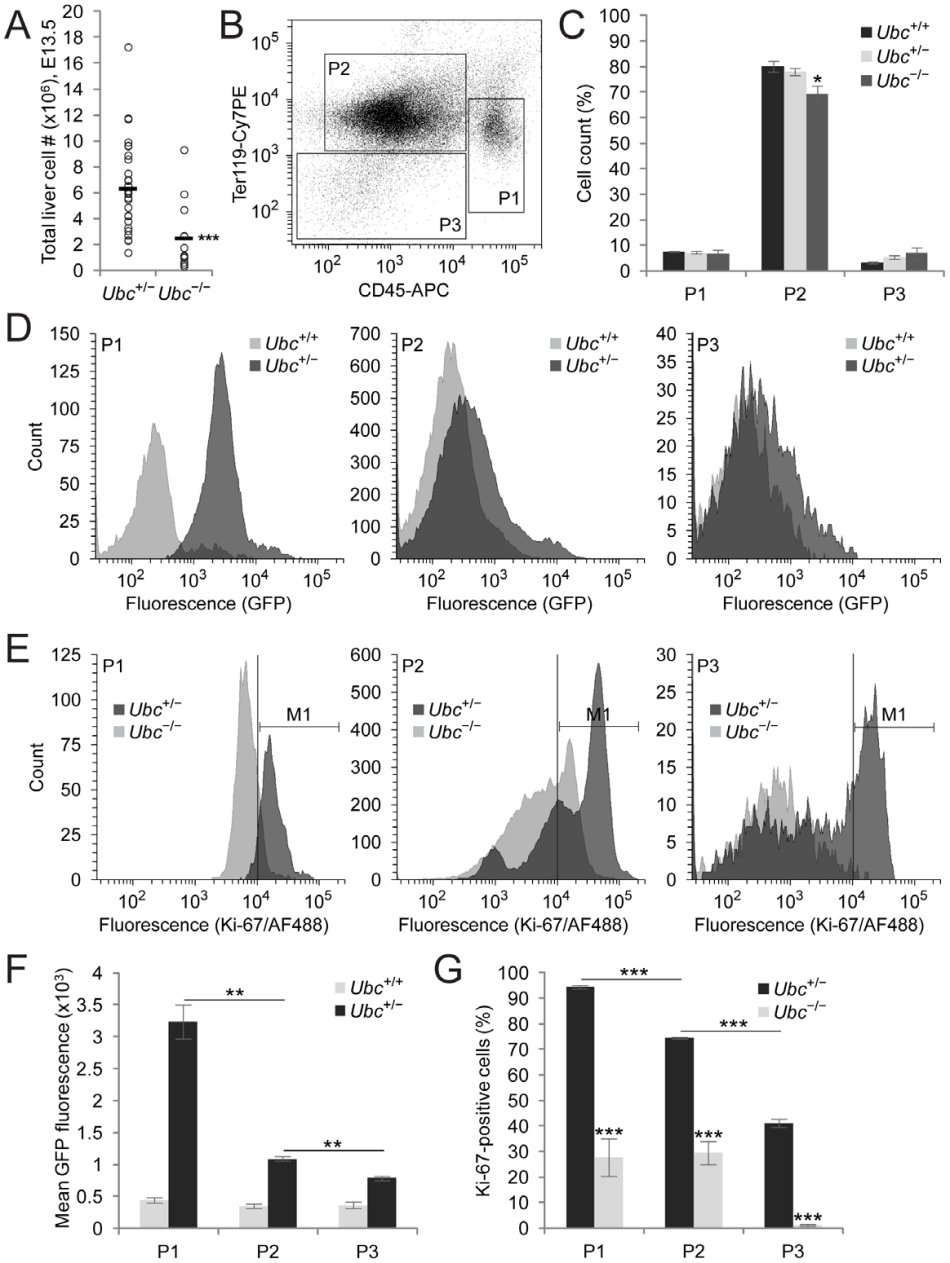
Increased *Ubc* expression and proliferation capacity in hematopoietic cells. (A) The number of total liver cells from *Ubc^+/−^* (*n* = 38) and *Ubc^−/−^* embryos (*n* = 13) at E13.5 was measured using a hematocytometer. (B) Fetal liver cells from *Ubc^+/−^* embryos at E13.5 were stained with CD45 and Ter119, and analyzed by flow cytometry. CD45^+^, Ter119^+^, and CD45*^−^*/Ter119*^−^* cells were defined as P1, P2, and P3 populations, respectively. (C) The percentage of P1, P2, and P3 populations in E13.5 *Ubc^+/+^*, *Ubc^+/−^*, and *Ubc^−/−^* fetal liver cells (*n* = 3 each) are shown. (D) Representative histograms for GFP fluorescence of P1, P2, and P3 populations in E13.5 *Ubc^+/+^* and *Ubc^+/−^* fetal liver cells. *Ubc^+/+^* cells served as background fluorescence controls because they were lack of GFP. (E) Representative histograms for Ki-67 immunoreactivity of P1, P2, and P3 populations in E13.5 *Ubc^+/−^* and *Ubc^−/−^* fetal liver cells. Ki-67 immunoreactivity was indirectly monitored by Alexa Fluor 488-conjugated anti-mouse IgG (AF488). Cells within the M1 marker area were considered as Ki-67-positive. (F) Mean GFP fluorescence of P1, P2, and P3 populations in E13.5 *Ubc^+/+^* and *Ubc^+/−^* fetal liver cells (*n* = 3 each). (G) The percentage of Ki-67-positive cells of P1, P2, and P3 populations in E13.5 *Ubc^+/−^* and *Ubc^−/−^* fetal liver cells (*n* = 3 each). All data in (A), (C), (F), and (G) are expressed as the means ± SEM from the indicated number of samples. In (A), open circles represent data from an individual sample and horizontal solid bars represent the means from the indicated number of samples. ^*^ *P*<0.05; ^**^ *P*<0.01; ^***^ *P*<0.001 *vs.* control (*Ubc^+/+^* or *Ubc^+/−^*) embryos unless otherwise indicated by horizontal bars.

To identify the cell types that exhibit high *Ubc* expression and the cell types that show altered proliferation capacity upon deletion of *Ubc*, we processed fetal liver cells, stained with CD45 and Ter119, and analyzed by flow cytometry. We were able to identify CD45-positive cells in both *Ubc^+/−^* and *Ubc^−/−^* fetal liver cells, which were present at similar frequencies of about 7% (Figure 3C). Similarly, we found that fetal liver-derived progenitor cell frequency was about 2% in both *Ubc^+/−^* and *Ubc^−/−^* fetal liver cells (data not shown). Because total liver cellularity was reduced by about 60% in the *Ubc^−/−^* fetal liver relative to *Ubc^+/−^* fetal liver (Figure 3A), these results indicate the concomitant reduction in the absolute number of hematopoietic cells and HSCs/progenitor cells in the *Ubc^−/−^* fetal liver. Ter119-positive cells were most abundant in both *Ubc^+/−^* and *Ubc^−/−^* fetal liver cells, although its frequency was reduced by about 10% in *Ubc^−/−^* fetal liver cells (Figure 3C). We then monitored GFP fluorescence of these cell populations in *Ubc^+/−^* fetal liver cells, and found that CD45-positive cells exhibited the highest GFP fluorescence, suggesting that *Ubc* expression levels in hematopoietic cells were much higher than any other cell types (Figures 3D and 3F). We also demonstrated that CD45-positive cells were highly proliferating and the percentages of Ki-67-positive proliferating cells were significantly reduced in *Ubc^−/−^* fetal liver cells regardless of the cell types (Figures 3E and 3G), which is consistent with the immunostaining results (see Figure 1C). Therefore, our data suggest that CD45-positive hematopoietic cells have the highest *Ubc* transcriptional activity and proliferation capacity in E13.5 embryonic liver, and there is a correlation between *Ubc* transcriptional activity and proliferation capacity among different cell types.

### The capacity of hematopoiesis in *Ubc^−/−^* fetal liver cells was only slightly affected under competitive microenvironment

**Figure 4:**
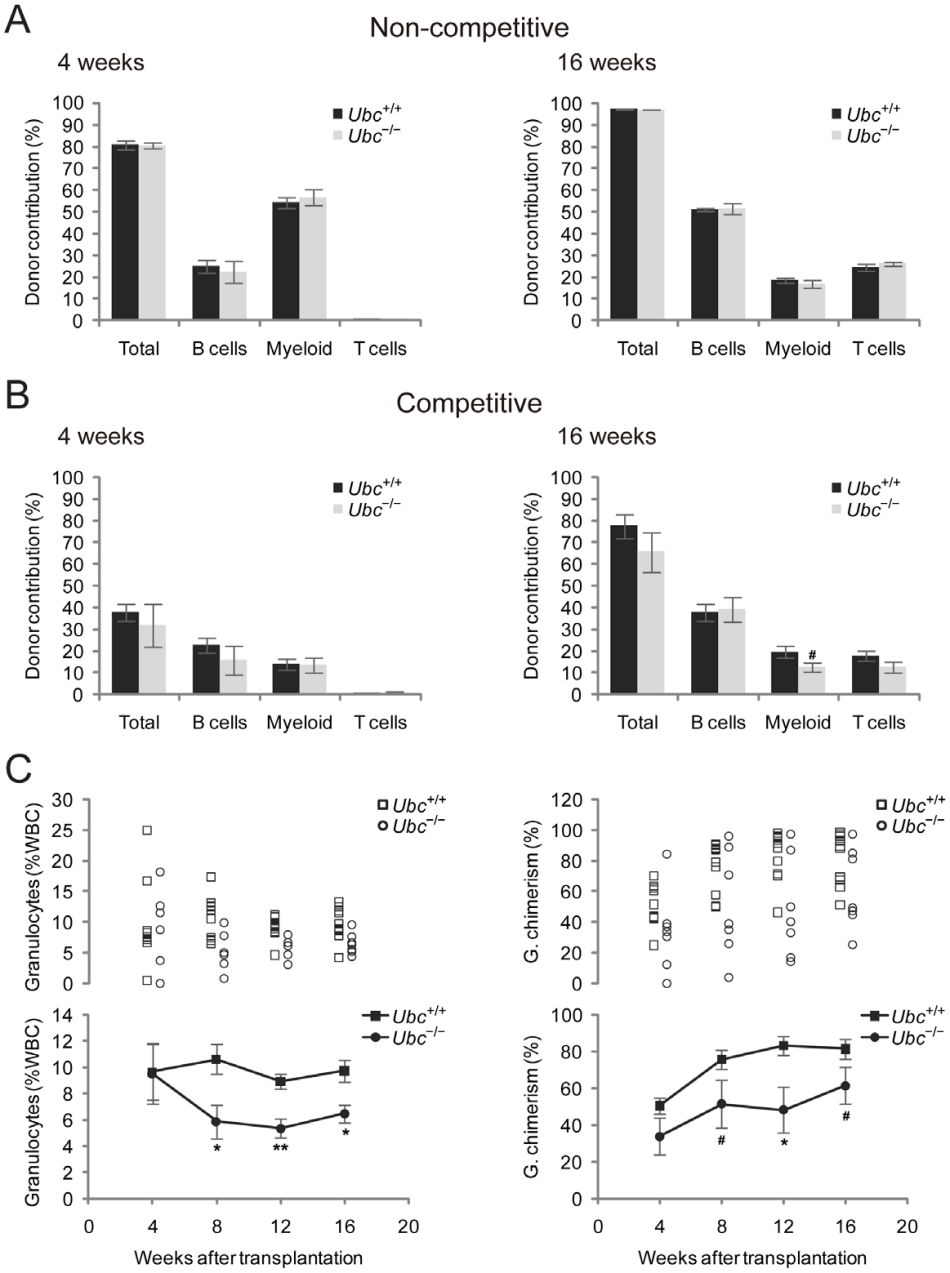
Only slightly reduced progenitor and HSC function in *Ubc^−/−^* fetal liver cells under competitive microenvironment. (A) For non-competitive reconstitution, lethally irradiated recipient mice were transplanted with whole fetal liver cells from E13.5 *Ubc^+/+^* and *Ubc^−/−^* embryos (*n* = 3 each). For each genotype, whole fetal liver cells from one embryo were used for transplantation into 3 recipient mice. (B) For competitive reconstitution, lethally irradiated recipient mice were transplanted with whole fetal liver cells from E13.5 *Ubc^+/+^* (*n* = 10) and *Ubc^−/−^* embryos (*n* = 7) together with recipient-type whole BM cells. Whole fetal liver cells from three different *Ubc^+/+^* embryos were used for transplantation into 13 recipient mice (3–5 recipient mice/embryo), but 3 out of 13 were not graphed because they were not multilineage engrafted. Similarly, whole fetal liver cells from three different *Ubc^−/−^* embryos were used for transplantation into 14 recipient mice (4–5 recipient mice/embryo), but 7 out of 14 were not graphed because they were not multilineage engrafted. In both reconstitution experiments, contributions of donor-marked progenitor/HSCs to lymphoid (B cells, T cells) and myeloid lineages were analyzed using peripheral blood drawn at 4 and 16 weeks after transplantation. (C) (Left panel) Donor-marked granulocyte frequency was expressed as % of white blood cells (%WBC) at 4-week intervals after transplantation of whole fetal liver cells from E13.5 *Ubc^+/+^* (*n* = 11) and *Ubc^−/−^* embryos (*n* = 7) into recipient mice in a competitive manner. (Right panel) Donor granulocyte chimerism was determined as described in Materials and Methods. All data in (A) and (B) are expressed as the means ± SEM from the indicated number of samples. In (C), open squares and circles represent data from an individual sample and closed squares and circles represent the means ± SEM from the indicated number of samples. ^#^ *P*<0.1; ^*^ *P*<0.05; ^**^ *P*<0.01 *vs.* wild-type (*Ubc^+/+^*) embryos.

At the midgestation period, HSCs migrate from the AGM into the liver, and the liver becomes the major site of hematopoiesis [18]. During this period, fetal liver cells proliferate extensively. We hypothesized that the reduced proliferation capacity in *Ubc^−/−^* fetal liver may hamper the function of HSC to support hematopoiesis, which can be detrimental to embryonic development. Therefore, we decided to examine whether impaired HSC function could be a cause for *Ubc^−/−^* embryonic lethality. In order to test whether the abnormality in the *Ubc^−/−^* fetal liver results in cell autonomous defects in HSC function, we assayed the progenitor/HSC activity using the non-competitive and competitive reconstitution method. For non-competitive reconstitution, 1×10^6^ whole fetal liver cells obtained from E13.5 embryos were transplanted into lethally irradiated mice (Figure 4A). For competitive reconstitution, 1.5×10^5^ fetal liver cells were transplanted together with 2×10^5^ congenic recipient-type bone marrow cells (Figure 4B). At 4, 8, 12, and 16 week after transplantation, peripheral blood cells from recipient mice were analyzed to examine the contribution of donor cells to lymphoid (T cells and B cells) and myeloid lineages (data not shown for 8 and 12 weeks). In both competitive and non-competitive settings, loss of *Ubc* had only a modest impact on HSC function both in terms of repopulation kinetics and total reconstitution of B cell, T cell, and myeloid lineages (Figures 4A and 4B). However, the long-term contribution to myeloid lineages was significantly diminished in the *Ubc^−/−^* competitive transplants at 16 weeks after transplantation.

To explore this further, we monitored donor-marked granulocyte reconstitution, which, because of their short life span (approximately 4–6 days) in contrast to T cells and B cells, has been used as a measure of an on-going HSC activity [22], [23]. Therefore, continued stem cell activity is required to keep generating granulocytes. Both granulocyte frequencies (Figure 4C, left panel) and granulocyte chimerism (Figure 4C, right panel) were diminished in the absence of *Ubc*, suggesting subtle autonomous defects in *Ubc^−/−^* HSCs. However, because the overall reconstitution of *Ubc^−/−^* HSCs was generally comparable to control cells, we can conclude that the *Ubc^−/−^* embryonic lethality may not be directly caused by cell autonomous failure in HSCs, although we cannot exclude the possibility that the reduced number of HSCs may play a role in embryonic lethality.

## Discussion

Disruption of the polyubiquitin gene *Ubc* leads to a defect in fetal liver development, which can be partially rescued by increasing the amount of ubiquitin (Ub) [13]. However, it is still not known why *Ubc* is required for fetal liver development and the nature of defective cell types that are responsible for the lethality in *Ubc^−/−^* embryos have not yet been characterized. At embryonic days (E) 11–11.5, right before the onset of *Ubc^−/−^* embryonic lethality, hematopoietic stem cells (HSCs) migrate into the liver, which rapidly enlarges and becomes the principal site of hematopoietic activity [18]. Therefore, it is possible that the cause of *Ubc^−/−^* embryonic lethality may be due to the failure in the fetal liver hematopoietic system.

In this study, we tried to identify the cell-type specific role of *Ubc* in fetal liver development, which cannot be compensated for by other ubiquitin genes. To this end, we first monitored the proliferation capacity in *Ubc^−/−^* fetal liver cells and found that it was significantly reduced and associated with increased apoptosis. Specifically, we found that *Ubc* was most highly expressed in hematopoietic cells, and the proliferation capacity of hematopoietic cells was significantly impaired in *Ubc^−/−^* embryos. Based on flow cytometric analysis, *Ubc* transcriptional activity seemed to correlate well with the proliferation status of the cells (see Figures 3F and 3G). These results suggest that upregulation of *Ubc* to maintain enough Ub pools may be required for cell proliferation. Therefore, cells with high *Ubc* transcriptional activity are highly proliferative with enough Ub pools. However, it is also possible that cells with high *Ubc* transcriptional activity are simply under stress regardless of their proliferation status or cell types.

All of the polyubiquitin gene knockout phenotypes seem to be related to cell-type specific reduction of Ub levels. For example, since *Ubc* was highly expressed in MEFs, Ub levels were reduced by ∼40% in *Ubc^−/−^* MEFs [13]. We demonstrated that reduced proliferation, premature senescence, and abnormal cell cycle progression in *Ubc^−/−^* MEFs were completely rescued by increasing the Ub levels up to wild-type levels [13]. Since *Ubb* is highly expressed in germ cells, Ub levels were dramatically reduced by ∼70% in adult *Ubb^−/−^* testes, which is mostly comprised of germ cells [14]. Interestingly, although Ub levels were not significantly reduced in *Ubb^−/−^* ovaries, they were reduced by ∼70% in isolated oocytes from *Ubb^−/−^* mice, suggesting that the effect on total Ub levels due to disruption of polyubiquitin gene depends highly on the contribution of the polyubiquitin gene to the total Ub pools, which varies based on cell types [14]. Hypothalamic phenotypes in *Ubb^−/−^* mice also seemed to be closely related to the reduced Ub levels in the hypothalamus by ∼30%, but not in the whole brain [15]. Therefore, reduced proliferation of *Ubc^−/−^* fetal liver cells is likely caused by the Ub deficiency in the *Ubc^−/−^* embryonic liver, in which Ub levels were reduced by ∼40%. Currently, it is not clear which cell types in the embryonic liver are affected most due to deletion of *Ubc*, although we speculate that hematopoietic cells may be affected most, simply based on their high *Ubc* expression levels. This type of analysis would require the isolation of specific cell types and determination of Ub levels therein.

Interestingly, whereas the frequency of primitive HSCs and hematopoietic cells were maintained (about 2% and 7% of total liver cells, respectively), the reduced cellularity of the *Ubc^−/−^* fetal livers lead to a reduction in the absolute number of HSCs and hematopoietic cells. We next assessed the cause of *Ubc^−/−^* embryonic lethality with respect to the failure in the fetal liver hematopoietic system. To investigate whether the hematopoietic defect is caused by the loss of *Ubc* during embryonic development, we transplanted whole fetal liver cells from wild-type and *Ubc^−/−^* E13.5 embryos into lethally irradiated recipient mice by non-competitive and competitive transplantation, and performed peripheral blood analysis to measure donor contribution to hematopoietic lineages at 4, 8, 12, and 16 weeks after transplantation. Under non-competitive conditions, the donor contribution to lymphoid and myeloid lineages was not significantly different between the two genotypes, suggesting that the hematopoietic function of *Ubc^−/−^* fetal liver remained intact. However, under competitive conditions, *Ubc^−/−^* fetal liver exhibited a marginal yet significantly reduced capacity to reconstitute hematopoietic function. Therefore, these combined results suggest that although the hematopoietic system in *Ubc^−/−^* embryos is slightly impaired, the embryonic lethality of *Ubc^−/−^* mice is not likely caused by an autonomous failure of the hematopoietic system.

Although *Ubc^−/−^* embryonic lethality may be caused by the reduced number of HSCs and hematopoietic cells with impaired proliferation capacity, our data also suggest that defects in erythroid cell development may also contribute to the *Ubc^−/−^* embryonic lethality. Although *Ubc* expression levels in Ter119-positive cells were not as high as CD45-positive cells (see Figure 3F), many Ter119-positive cells were apoptotic and the proliferation capacity of Ter119-positive cells was also significantly impaired in *Ubc^−/−^* fetal liver (see Figure 3G). These were reflected in the reduced frequency of Ter119-positive cells in *Ubc^−/−^* fetal liver cells (see Figure 3C). In addition, as shown in our previous report [13], the color of *Ubc^−/−^* fetal liver was very pale and the close examination of fetal liver histology revealed the reduced number of mature enucleated red blood cells, which were stained intensely red with H&E, in *Ubc^−/−^* fetal liver. Taken together, it is also possible that *Ubc^−/−^* embryonic lethality could be caused, at least in part, by defects in erythroid cell development, possibly resulting in anemia. Further detailed analysis will prove the cell autonomous and non-cell autonomous role of *Ubc* in erythroid cell development.

In conclusion, *Ubc^−/−^* embryonic lethality does not seem to be due to intrinsic failure of the hematopoietic system, but rather may be due to the reduced number of HSCs. It is also possible that *Ubc^−/−^* embryonic lethality could, at least in part, be due to defects in the proliferation and/or differentiation capacity of fetal liver epithelial cells such as hepatocytes and ductal cells. Further investigation using these cell types is required to directly prove the role of Ub in fetal liver development and eventually identify the underlying molecular mechanisms for the embryonic lethality of *Ubc^−/−^* fetal liver.

## Materials and Methods

### Mouse studies

All mice were maintained in plastic cages with *ad libitum* access to food and water. All animal procedures followed National Institute of Health guidelines with the approval of Stanford University Administrative Panel on Laboratory Animal Care (APLAC; #13745) and the University of Seoul Institutional Animal Care and Use Committee (UOS IACUC; UOS-091201-1).

### Immunostaining and TUNEL assay

For immunohistochemistry, embryos were isolated and fixed in 4% paraformaldehyde overnight at RT, washed with 70% ethanol, dehydrated, and embedded in paraffin. Sagittal embryonic sections (4 µm thick) were prepared using a microtome, deparaffinized, rehydrated, and stained with anti-Ki-67 mouse monoclonal antibody (1∶20, BD Pharmingen) using Histomouse™-SP kit (Zymed) according to the manufacturer's protocols and visualized with a Zeiss Axio Imager microscope. For immunofluorescence, embryos were isolated and fixed in cold 4% paraformaldehyde for 4 hours with gentle rocking, cryoprotected with 30% sucrose, and embedded in OCT freezing medium. Thaw-mounted sagittal embryonic sections (7 µm thick) were generated using a cryostat, permeabilized with 0.3% Triton X-100/PBS, and blocked with 3% BSA/0.1% Triton X-100/PBS for 1 hour at RT. Embryonic sections were incubated with anti-phospho-histone 3 (PH3) rabbit polyclonal antibody (1∶200, Upstate) at 4°C overnight, washed with 0.1% Triton X-100/PBS, and incubated with Alexa Fluor 555-conjugated goat anti-rabbit IgG (1∶200, Invitrogen) and TO-PRO-3 iodide (1∶1,000, Invitrogen) for 1 hour at RT. Sections were washed with 0.1% Triton X-100/PBS, PBS only, and then mounted using the ProLong Gold antifade reagent (Invitrogen). A confocal microscope was used as described previously [15], [16].

The TUNEL (terminal deoxynucleotidyl transferase dUTP nick end labeling) assay was performed using an Apoptag® peroxidase *in situ* apoptosis detection kit (Chemicon) for paraffin-embedded sections, and an Apoptag® red *in situ* apoptosis detection kit (Chemicon) for thaw-mounted frozen sections according to the manufacturer's protocols.

### Cytospin of fetal liver cells

Fetal liver tissues were pulverized in PBS containing 1 mM EDTA and 1% fetal bovine serum, treated with HBSS containing 1 mg/ml collagenase, 1 mg/ml hyaluronidase, and 12.5 µg/ml DNase I for 30 min at 37°C, and medium containing 10% fetal bovine serum was added before cytospin at 800 rpm for 5 min. Equal numbers of fetal liver cells were used to attach on a cytospin slide (20,000 cells/slide). Cells on cytospin slides were stained as described previously with some modifications [13]. Briefly, cells were fixed in 4% paraformaldehyde for 10 min at RT, permeabilized with 0.2% Triton X-100/PBS, and blocked with 0.5% BSA/PBS for 30 min at RT. Fixed cells were incubated with anti-CD45 rat monoclonal antibody (1∶20, BD Pharmigen), anti-E-cadherin mouse monoclonal antibody (1∶20, BD Pharmigen), anti-alpha-fetoprotein (AFP) rabbit polyclonal antibody (1∶400, Thermo Scientific), or anti-Ter119 rat monoclonal antibody (1∶500, BD Pharmigen) in combination with anti-Ki-67 mouse monoclonal antibody (1∶100, BD Pharmingen) or anti-phospho-histone 3 (PH3) rabbit polyclonal antibody (1∶100, Upstate) at 4°C overnight, washed with PBS, and incubated with appropriate Alexa Fluor 488 or 555-conjugated goat anti-mouse, rat, or rabbit IgG (1∶400, Invitrogen) for 1 hour at RT. Cells were mounted using the ProLong Gold antifade reagent with DAPI (Invitrogen) and visualized with a Zeiss Axio Imager microscope. To detect apoptotic cells, the double-labeling fluorescence TUNEL assay was performed using Apoptag® red *in situ* apoptosis detection kit with appropriate markers according to the manufacturer's protocols.

### Flow cytometric analysis of fetal liver cells

Fetal liver tissues were pulverized in PBS containing 1 mM EDTA and 1% fetal bovine serum, and total liver cell number was counted using a hematocytometer. Isolated fetal liver cells were fixed in 4% paraformaldehyde, permeabilized with 90% methanol, and stained with anti-CD45-APC antibody (1∶200, BD Pharmigen, pan-hematopoietic marker) and anti-Ter119-Cy7PE antibody (1∶1000, BD Pharmigen, erythroid cell marker). To detect cells in proliferation, fetal liver cells were co-stained with anti-Ki-67 antibody (1∶50, BD Pharmingen), followed by Alexa Fluor 488-conjugated anti-mouse IgG (1∶400, Invitrogen). Isotype control or secondary antibody was used to determine the range of background fluorescence. Using FACSCanto II (BD Biosciences), 5×10^4^ events were collected and gated for CD45^+^, Ter119^+^, CD45*^−^*/Ter119*^−^* cells to obtain the frequency of specific cell types.

### Transplantation and HSC/progenitor reconstitution analysis

#### Transplantation

Recipient mice (CD45.1) were lethally irradiated with a dose of 800 rad and whole fetal liver cells from donor (CD45.2) were obtained from E13.5 embryos. For non-competitive transplantation, 1×10^6^ cells were transplanted by tail vein within 6 hours of irradiation. For competitive transplantation, 1.5×10^5^ cells were transplanted by tail vein with 2×10^5^ recipient-type whole bone marrow (BM) cells.

#### Reconstitution analysis

Reconstituted mice were periodically bled via tail vein to monitor contribution by donor-marked HSC/progenitors in the peripheral blood of B, T, and myeloid lineages at 4, 8, 12, and 16 weeks post-transplant. Using a heat lamp, the tail was heated and a sharp incision was made to collect about 4–6 drops of blood in a tube containing 10 mM EDTA/PBS. An equal volume of 2% dextran/PBS was added to generate a density gradient and incubated at 37°C for 30 min to precipitate red blood cells. The supernatant was collected and the remaining red blood cells were lysed in buffer containing ammonium chloride and potassium bicarbonate. Cells were then stained with anti-CD45.1-PE antibody (to detect recipient cells), anti-CD45.2-FITC antibody (to detect donor cells), anti-Ter119-Cy5PE antibody (erythroid cell marker), anti-B220-Cy7APC antibody (B lymphocyte marker), anti-CD3-APC antibody (T lymphocyte marker), and anti-Mac1-Cy7PE antibody (myeloid cell marker). Using LSR II (BD Biosciences), 1×10^6^ events were collected and Ter119^−^ cells (also PI^−^ for live cells) were gated for donor cells (CD45.2^+^) and analyzed for B cells (B220^+^) *vs.* myeloid cells (Mac1^+^, including Mac1^high^ and Mac1^low^) in donor cell populations. B220^−^ and Mac1^−^ populations were further analyzed for T cells (CD3^+^). As reported previously, T cells were hardly present in donor cell populations at 4 weeks post-transplant [24].

Donor-marked granulocyte frequency was expressed as % of white blood cells (WBC) at 4-week intervals after transplantation into recipient mice. Donor granulocyte chimerism was determined by analyzing the percentage of Ter119^−^CD3^−^B220^−^Mac1^high^side scatter^high^ cells that were also donor^+^.

### Indirect competitive ELISA and quantitative real-time RT-PCR

Indirect competitive ELISA and quantitative real-time RT-PCR were carried out as described previously [13], [15].

### Statistical analysis

Two-tailed unpaired Student's *t*-tests were used to compare the data between two groups. Although *P*<0.05 was considered to be statistically significant in most cases, *P*<0.1 was also noted in certain cases.
